# International Solutions for Continual Gaps in LGBTQ + Education and Exposure

**DOI:** 10.1007/s10900-024-01384-z

**Published:** 2024-07-28

**Authors:** Dustin Z. Nowaskie, Samuel D. Garrison

**Affiliations:** 1 Department of Psychiatry and the Behavioral Sciences, Keck School of Medicine of University of Southern California, 1031 W. 34th St, Los Angeles, CA 90089 USA; 2 Department of Psychiatry, Indiana University School of Medicine, Indianapolis, IN USA

It is well-established that lesbian, gay, bisexual, transgender, queer, and all sexual and gender diverse (LGBTQ+) communities experience alarming and disproportionately higher health disparities compared to cisgender, heterosexual populations. Origins of LGBTQ + disparities are multifaceted, occur throughout life, and are rooted in adverse childhood experiences, traumas, and stigmas (including social, political, and healthcare marginalizations). For instance, LGBTQ + youth are often at high risk for bullying, assault, poor academic performance, unsafe sexual behaviors, homelessness, sexually transmitted infections, depression, anxiety, substance use, and suicidality [1]. Likewise, LGBTQ + adults experience similar healthcare disparities in addition to chronic, physical health conditions (e.g., pain, heart diseases, cognitive decline, and cancers) [2].

Because of pervasive discrimination and victimization, LGBTQ + youth and adults are often faced with difficult choices of existing in non-affirming spaces (e.g., their homes and communities) while “in the closet”, being “out” only in particular contexts, or living in accordance with their true identities yet succumbing to stigmas, all of which are largely traumagenic and further exacerbate health factors and outcomes [1]. These realities for LGBTQ + people extend into healthcare environments as well, which are at least partially secondary to biases, traumatic experiences, as well as lack of education, training, and patient exposure from health professional students, residents, and providers [3–8]. Consequently, paucities of LGBTQ + education and affirmation ultimately perpetuate poor patient-provider interactions, overall general mistrust in providers and medical communities, and LGBTQ + health disparities.

Currently, there are substantial international deficiencies in LGBTQ + educational content at all levels of health professional education and training programs [3–8]. Despite consistent recommendations from major health organizations to incorporate LGBTQ + topics into curricula, there has been minimal progress in current health professional training programs to increase the time devoted to LGBTQ + education [9]. A recent study revealed that medical students receive two annual curricular hours of LGBTQ + content, reflecting a modicum of improvement from studies conducted nearly a decade prior [6]. Similar studies surveying residents across multiple specialties (e.g., dermatology [8], plastic surgery [4], psychiatry [3], and urology [5]) have revealed insufficient deliveries of LGBTQ + education (often, one to three annual hours).

While it is imperative that efforts must be mobilized to increase the overall attention to LGBTQ + care within health professional education and training programs, consideration of several explicit themes should also be noted. Firstly, as the social contexts of LGBTQ + communities are continually evolving to encompass a wider understanding of diverse identities, education must be longitudinal to adapt to rapid changes in these terminologies and understandings to assure affirming care. Secondly, LGBTQ + education cannot be singular nor general, but must start foundational and then supplemented with diverse topics in primary care, mental health, gender-diverse care, and many more. Past studies consistently indicate topic-specific differences, i.e., students, residents, and providers receiving notably less gender diverse education, experience, and supervision compared to sexual diverse topics [3–8]. Thirdly, LGBTQ + patient exposure is vital, as care for more LGBTQ + patients is associated with students, residents, and providers feeling more adequately affirming to care for LGBTQ + people [3–8]. Furthermore, while relatively new, some accrediting bodies have diverse populations requirements, specifying that learners and providers must demonstrate competency caring for diverse patient populations (i.e., diversity in gender, age, race, sexual orientation, etc.), but they do not require specific LGBTQ + content [9]. To ensure the best quality of LGBTQ + care and equity, LGBTQ + education and patient exposure must be first introduced within health professional schools [6] to institute a foundation of knowledge and preparedness and then supplemented longitudinally through multimodal integrations within residency programs, institutions, systems, and beyond [3–5, 8, 9].

Future efforts to transform LGBTQ + healthcare should emphasize holistic learning and experience through multidisciplinary and multidimensional lenses. Currently, many health professional training programs do not have infrastructures, staff, or diversity, equity, and inclusion initiatives to provide the necessary amount of education and patient exposure. However, solutions do exist for institutions and systems to utilize external resources to supplement and fortify their LGBTQ + exposure. OutCare Health is a leading international nonprofit 501(c)(3) LGBTQ + organization dedicated to promoting LGBTQ + care and equity worldwide via affirming information, education, resources, support, public health, community building, advocacy, and research [10]. OutCare Health delivers a multitude of information, resources, referrals, programming, and educational modules for healthcare institutions and systems to integrate LGBTQ+  topics and improve knowledge, skills, and strategies amongst students, residents, providers, and all healthcare staff. For example, OutCare Health’s The National LGBTQ + Curriculum is a comprehensive library of on-demand LGBTQ + educational and training modules led by diverse people; Part I is an 8-module foundational series covering terminology, communication, mental health, and primary care, while Part II is a 14-module series spanning specific gender diverse topics. The National LGBTQ + Curriculum has been widely well received by thousands of participants, indicating a 95+% satisfaction rate. Additionally, OutCare Health offers a variety of one-hour specialized training seminars. Healthcare students, providers, and staff can also increase their LGBTQ + exposure via OutCare Health’s free community initiatives including the OutList affirming provider directory, OutTalk series on diversity and intersectionality, OutReach support groups, OutPost blogs, Mentorship Program, Community Resource Database, LGBTQ + research, and much more [10]. Through these LGBTQ + initiatives, all healthcare institutions and systems can enhance and amplify their LGBTQ + attention and affirmation to deliver the highest quality of LGBTQ + care.

The current state of LGBTQ + education and exposure in healthcare is evidently insufficient in addressing longstanding, pervasive health disparities within LGBTQ + communities. While future advocacy efforts must be positioned to demand accrediting organizations to validate LGBTQ + identities and mandate LGBTQ + education, it is abundantly encouraging that more younger generations of healthcare providers remain hopeful, intentional, and committed in providing equitable and affirming care to LGBTQ + people. The immediate step herein lies a call to action to all institutions and systems to rectify the current lack of LGBTQ + attention by elevating the next generation of providers and equipping learners with the necessary knowledge and tools to provide LGBTQ + patients with safe, compassionate, and unbiased care of which they are deserving and long overdue. By engaging international community organizations, institutions may begin taking these paramount steps in the right direction towards a future free of biases, discriminations, and prejudices against LGBTQ + communities in healthcare systems.

## Data Availability

Not applicable.

## Declarations

### Authors’ Information

The author DZN is the Founder and President of an organization referenced in this paper, OutCare Health, which is a nonprofit, 501(c)(3) LGBTQ + health equity organization.
